# Chimeric Antigen Receptor (CAR)-T Cell Therapy for Non-Hodgkin's Lymphoma

**DOI:** 10.20411/pai.v9i1.647

**Published:** 2024-03-15

**Authors:** Maria Florencia Giraudo, Zachary Jackson, Indrani Das, Olubukola M. Abiona, David N. Wald

**Affiliations:** 1 Department of Pathology, Case Western Reserve University, Cleveland, Ohio; 2 Department of Pathology, Louis Stokes Cleveland VA Medical Center, Cleveland, Ohio

**Keywords:** Cell Therapy, Lymphoma, Immunotherapy, CAR-T, Chimeric Antigen Receptors

## Abstract

This review focuses on the use of chimeric antigen receptor (CAR)-T cell therapy to treat non-Hodgkin's lymphoma (NHL), a classification of heterogeneous malignant neoplasms of the lymphoid tissue. Despite various conventional and multidrug chemotherapies, the poor prognosis for NHL patients remains and has prompted the utilization of groundbreaking personalized therapies such as CAR-T cells. CAR-T cells are T cells engineered to express a CAR that enables T cells to specifically lyse tumor cells with extracellular expression of a tumor antigen of choice. A CAR is composed of an extracellular antibody fragment or target protein binding domain that is conjugated to activating intracellular signaling motifs common to T cells. In general, CAR-T cell therapies for NHL are designed to recognize cellular markers ubiquitously expressed on B cells such as CD19^+^, CD20^+^, and CD22^+^. Clinical trials using CAR-T cells such as ZUMA-7 and TRANSFORM demonstrated promising results compared to standard of care and ultimately led to FDA approval for the treatment of relapsed/refractory NHL. Despite the success of CAR-T therapy for NHL, challenges include adverse side effects as well as extrinsic and intrinsic mechanisms of tumor resistance that lead to suboptimal outcomes. Overall, CAR-T cell therapies have improved clinical outcomes in NHL patients and generated optimism around their future applications.

## NON-HODGKIN'S LYMPHOMA

Non-Hodgkin's lymphoma (NHL) is a classification of malignant neoplasm of the lymphoid tissue that involves white blood cells, specifically, precursors for B and T cells and their mature counterparts [1]. While NHL is the seventh most common malignancy in the United States, it is an umbrella term for a myriad of subtypes, such as Burkitt lymphoma, primary CNS lymphoma, mycosis fungoides, and marginal zone lymphoma, with the most common types being mantle cell lymphoma, follicular lymphoma, and diffuse large B cell lymphoma [1, 2].

Approximately 4% of cancer diagnoses in the United States are of NHL, with an increase in prevalence of 168% since 1975 [2]. Even with conventional treatment or multidrug chemotherapies, regimens for NHL are often unsatisfactory and [3, 4] typically yield a poor prognosis. This increase in incidence and low efficacy of conventional therapies has led to the pioneering research of novel therapies including immunomodulatory agents, B-cell receptor (BCR) signaling inhibitors, epigenetic modulators, monoclonal antibodies (mAbs), Bcl-2 inhibitors, checkpoint inhibitors, and adoptive T-cell therapy [5–13]. Despite the various treatment types, up to 50% of patients have relapsed or become refractory after treatment for diffuse large B-cell lymphoma (DLBCL), highlighting the need for more personalized therapies such as chimeric antigen receptor (CAR)-T cells [14, 15]. In this review, we focus on the various facets of the application of CAR-T cells in NHL patients.

## THE STRATEGY OF CAR-T CELL THERAPY

**Figure 1.**
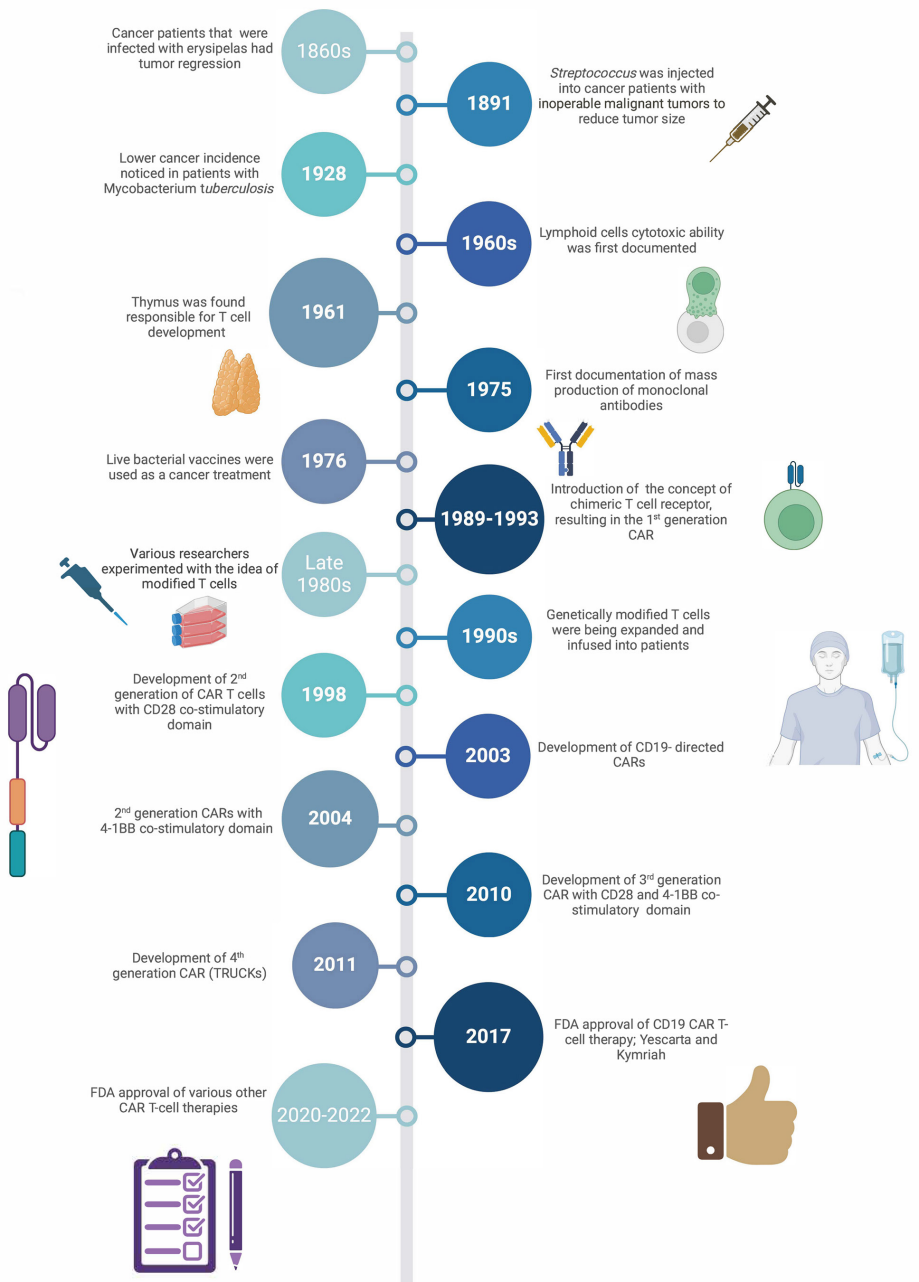
**Timeline of scientific and clinical milestones of the development of CAR-T cell therapy.** 1860s – German physicians Dr(s). Friedrich Fehleisen and Wilhelm Busch independently noted that cancer patients infected with erysipelas experienced tumor regression [22, 23]. 1891 – Bone surgeon Dr. William B. Coley successfully reduced the size of inoperable malignant tumors by injecting *Streptococcus* into cancer patients. Dr. Coley's contributions earned him the title as the “Father of Immunotherapy” [24]. 1928 – Dr. Raymond Pearl noted that patients infected with Mycobacterium *tuberculosis* had a lower incidence of cancer [25]. 1960s – Transplant rejection experiments uncovered the cytotoxic ability of lymphoid cells [26, 27]. 1961-1962 – Dr. Jacques Miller identified the thymus as the organ responsible for T cell development [28]. 1975 – Dr(s). César Milstein and Georges J.F. Köhler first documented the production of monoclonal antibodies with a predefined specificity [29, 30]. 1976 – Bacillus Calmette-Guérin (BCG), a live-attenuated tuberculosis vaccine, was found effective in treating cancers such as superficial bladder tumors [31]. 1989-1993 – Dr. Zelig Eshhar introduced the concept of a chimeric T cell receptor, resulting in the 1^st^ generation CAR [16, 17]. 1980s – Various researchers such as Dr(s). Steven Rosenberg, Michel Sadelain, Carl June, and Dario Campana experimented with the idea of utilizing modified T cells as a therapeutic against cancer [32–42]. 1990s – June and Sadelain were able to optimally expand the genetically engineered T cells and infuse them into patients [43–49]. 1998 **–** Development of 2nd generation of CAR-T cells with CD28 co-stimulatory domain [18, 50]. 2003 – Development of CD19-directed CARs [51]. 2004 – Modified 2^nd^ generation CARs with 4-1BB co-stimulatory domain [52–54]. 2009 **–** Development of 3^rd^ generation CAR with CD28 and 4-1BB co-stimulatory domain [55–57]. 2011 – Development of 4^th^ generation CARs also known as TRUCKs [58]. 2017 **–** FDA approval of CD19 CAR-T cell therapies Yescarta (axicabtagene cilloleucel) and Kymriah (tisagenlecleuccel) for refractory or relapsed leukemia and lymphoma patients [18, 59, 60]. 2020-2022 – Various other CAR-T cell therapies have been approved by the FDA over years: Tecartus (Brexucabtagene autoleucel), Breyanzi (Lisocabtagene maraleucel), Abecma (Idecabtagene vicleucel), Carvykti (Ciltacabtagene autoleucel) [18, 19, 61–64]. Created in BioRender.com

Fundamental immunological discoveries around the 1800s contributed to broadening the understanding of T cells and their potential clinical utilization to treat disease (Figure 1). Between the 1980s and 1990s, Dr. Zelig Eshhar from the Weizmann Institute of Science pioneered the novel concept of chimeric antigen receptors [16, 17] that ultimately led to the design of the current CAR utilized therapeutically [18–21].

CAR-T therapy is a type of adoptive cell therapy that genetically engineers T cells to display receptors that drastically improve their cytotoxic activity when encountering tumor-associated antigen on cell surfaces. In unmodified T cells, activation is required prior to cytotoxic activity and occurs via T cell receptors (TCRs) binding to a peptide presented by major histocompatibility complex (MHC) molecules on professional antigen-presenting cells (ie, dendritic cells, macrophages). CD8^+^ T cells recognize peptides presented by class I (MHC-I) and CD4^+^ T cells by class II (MHC-II), respectively. Afterward, the T cells migrate to the tumor and bind to tumor cells that present cognate peptide with MHC and induce apoptosis [22]. In contrast, CAR-T cells function without requiring this initial activation or peptide presentation by MHC [23–26]. CAR-T cells overcome the obstacle of MHC restriction by displaying cell surface molecules incorporating synthetic antibodies or target protein binding domains that recognize antigens in an HLA-independent manner [26, 27]. CAR-T cells were initially tested in patients diagnosed with chronic lymphocytic leukemia and subsequently with patients afflicted by acute lymphoblastic leukemia [28]. Since then, CAR-T cells have been tested as a potential therapy against both tumor and non-tumor indications, such as autoimmune disorders [26, 29].

## CAR DOMAIN-STRUCTURE FUNCTION

CAR-T cells usually consist of a target antigen-binding domain, hinge sequence motif, a transmembrane domain, and one or more intracellular signaling domains [30, 31]. The target antigen-binding domain is most frequently derived from an antibody single-chain variable fragment (scFv) with heavy and light chains connected by a flexible linker. The hinge region, also known as the extracellular spacer, connects the transmembrane and antigen-binding domains and is responsible for the degree of flexibility of the CAR and its position on the membrane [32, 33]. The transmembrane domain contains hydrophobic alpha helices that traverse the plasma membrane and anchor the CAR on the cell surface. Interestingly, the transmembrane domain dictates the stability of the receptor at the plasma membrane. The CD28 transmembrane domain has been reported to confer the greatest stability on the receptor of transmembrane domains tested to date [29, 34]. The intracellular signaling domain typically contains a portion of the CD3 zeta chain (CD3ζ) with 3 immunoreceptor tyrosine-based activation motifs (ITAMs) as well as 1 or 2 costimulatory molecule motifs selected from the following options: 4-1BB (CD137), OX40 (CD134), CD27, CD28, glucocorticoid-induced tumor necrosis factor (TNF), and inducible costimulatory (ICOS; CD278) [29, 35]. The intercellular signaling domain is responsible for triggering downstream signaling cascades that can cause differentiation, cytokine production, cytotoxic response, and recruitment of other immune cell types [34–36].

## GENERATIONS OF CARs

CAR-T cells are commonly grouped into 4 generations. First-generation CARs only incorporate CD3ζ without additional intracellular signaling domains and have demonstrated poor clinical results due to a lack of persistence and poor activation of T cells despite their promising results *in vitro* [17, 32, 36, 37]. The lack of therapeutic effectiveness contributed to the addition of a secondary signal, which led to the development of second- and third-generation CARs that contain CD3ζ with 1 or 2 costimulatory domains, respectively [38]. Each generation demonstrated good T-cell persistence and activation *in vitro and has had clinical success* [39, 40]. Fourth-generation CARs also co-express additional genes such as cytokines, chemokines, or suicide genes that can enhance the efficacy and safety of the CAR-T products. Fourth-generation CARs have been tested that can secrete cytokines, including interleukin (IL)-7, IL-12, IL-15, IL-18, and IL-23. It is thought that in some cases, the production of cytokines can be used as a treatment for heterogeneous solid tumors due to their capacity to support the elimination of cancer cells through CAR-independent mechanisms [28, 29]. Due to safety concerns with CAR-T products killing non-cancerous cells, they can also be engineered to express suicide genes like iCaspase-9 to mitigate on-target off-tumor toxicity [41–45].

## CAR-T CELL THERAPIES IN NHL

The use of CAR-T cell therapy for NHL has focused primarily on aggressive B-cell NHL, one of the most common forms of NHL. As most B-cell malignancies express CD19, CAR constructs against CD19 were chosen. Clinical trials and mouse studies using these CD19 CAR-T cells have demonstrated promising results. Three large-scale multicenter phase 3 clinical trials, ZUMA-7, TRANSFORM, and BELINDA, have been carried out for patients with B-cell NHL, with each trial containing a different second-generation CAR construct. Both ZUMA-7 and BELINDA received FDA approval for CD19 CAR-T products axicabtagene ciloleucel (axi-cel) and tisagenlecleucel (tisa-cel), respectively.

**ZUMA-7:** ZUMA-7, a phase 3 international trial, focused on poor prognosis NHL patients with relapse occurring within the past 12 months or refractory disease after first-line anthracycline-based and rituximab chemoimmunotherapy. The study consisted of placing a total of 359 patients (median age 59 years) from 77 worldwide sites randomly into 2 groups either receiving investigator-selected standard-care chemoimmunotherapy (179 patients) or a single infusion of CAR-T cells intravenously (180 patients) [46]. Patients in the CAR-T therapy group received axi-cel, an autologous anti-CD19 second-generation CAR, at a dose of 2.0 x10^6^ CAR-T cells per kilogram of body weight. The axi-cel CAR construct contained a murine CD19-specific scFv, CD28-H, CD28-TM, CD28-CS, and CD3ζ [46–48]. Prior to CAR-T infusion, patients underwent leukapheresis at day -5 followed by pre-conditioning chemotherapy with cyclophosphamide and fludarabine at days -4 and -3. The dose of cyclophosphamide and fludarabine was 500 mg per square meter of body-surface area per day and 30 mg per square meter per day, respectively. Patients in the standard chemotherapy group received 2 or 3 cycles of platinum-based chemoimmunotherapy. Overall, axi-cel therapy showed significantly better results in event-free response and survival than standard care. The median event-free survival was 2.0 months and 8.3 months for the group that received standard-of-care therapy and axi-cel therapy, respectively. Moreover, 50% of standard-care patients had a partial response and 32% had a complete response, while 83% of axi-cel therapy patients had a response and 65% had a complete response [46].

**TRANSFORM:** TRANSFORM was an international phase 3 trial for B-cell NHL that was coordinated in 47 sites across Japan, Europe, and the United States. Patients enrolled in the trial had to have had relapsed or refractory disease within 12 months after initial therapy. Patients were also required to have organ function and an Eastern Cooperative Oncology Group performance status score of 1 or less. A total of 184 patients were assigned to receive either CAR-T cells intravenously (92 patients) or standard of care (92 patients). The standard care consisted of 3 cycles of salvage immunochemotherapy [49]. The patients received lisocabtangene maraleucel (liso-cel), a second-generation anti-CD19 CAR with a murine scFv, IgG4-H, CD28-TM, 4-1BB-CS, and CD3ζ [47–49]. Results demonstrated that the group receiving liso-cel had significantly improved event-free survival. Patients who received standard of care had 2.3 months of event-free survival, while patients receiving liso-cel had 10.1 months. Moreover, patients treated with liso-cel had a higher complete response rate (61 out of 92 patients) compared to standard-care patients (36 out of 92 patients) [49].

**BELINDA:** BELINDA was an international phase 3 trial for patients with aggressive B-cell NHL completed in 18 countries in over 65 centers. Participants enrolled in BELINDA had a relapse or refractory period within the past 12 months. After going through leukapheresis, a total of 322 patients were randomly divided into 2 groups, either standard care (160 patients) or the tisa-cel group (162 patients). The standard-care patients received autologous hematopoietic stem-cell transplantation (HSCT) or salvage chemotherapy [50]. The tisa-cel group received a second-generation CAR construct that contained a murine scFv, CD8α-H, CD8α-TM, 4-1BB-Cs, and CD3ζ [47].

Overall, results demonstrated that both the standard-of-care and tisa-cel groups had 3 months of event-free survival. In addition, there was a response rate of 46.3% in the tisa-cel group and 42.5% in the standard-of-care group. Thus, there was no significant difference between patients in the tisa-cel group and those in the standard-care group [50]. Of note, the tisa-cel CAR did show favorable results in the JULIET phase 2 trial as compared to historical outcomes with chemotherapy [51].

## LIMITATIONS TO CAR-T CELL THERAPY

While CAR-T therapies have proven to be highly efficacious, serious adverse side effects have been noted. Commonly reported on-target effects include cytokine release storm (CRS) and neurotoxicity. Although these incidents are well-documented, the causative factors are still not fully known.

CAR-T cells effectively eliminate tumor cells through the release of perforin, granzymes, and cytokines, such as IL-1, IL-6, IL-15, interferon-gamma (IFN-γ), and GM-CSF. Rapid activation and expansion of these T cells can lead to elevated levels of cytokines, creating a systemic inflammatory environment and resulting in a cytokine release storm (CRS), a life-threatening condition. In the phase 3 ZUMA-7 trial for refractory large B-cell lymphoma, CRS of any grade was noted in 92% of participating patients in the axi-cel treatment group, which received infused CAR-T cells [46]. In current clinical practice, the anti-IL-6 therapy tocilizumab has been successfully utilized to mitigate CRS and reduce its complications [52].

Neurotoxicity is a commonly associated adverse side-effect with all generations of CAR-T therapies, and the resulting syndrome is known as immune effector cell-associated neurotoxicity (ICANS). ICANS is associated with a broad spectrum of symptoms such as headaches, altered mental status, and seizures. While the underlying pathophysiology is still unknown, current theories point to secondary expression of the targeted antigen in other parts of the host. For example, evidence of CD19 CAR-T cells being detected in cerebral spinal fluid has led to a prevailing theory that the CAR-T cells, through an unknown mechanism, are infiltrating brain tissue and interacting with an undetermined population of CD19-expressing cells. It was only recently through RNA-Seq that cerebral CD19 expression was confirmed in brain mural cells [53]. Other theories suggest that systemic inflammation may also be responsible. Nevertheless, these observations and ideas provide insight into the origins of ICANS symptomatology. Typically, patients experiencing ICANS are treated with corticosteroids, which have been found to be effective in mitigating symptoms [52].

Another on-target toxicity of CAR-T products for NHL, specifically CD19 CAR-T cells, is B-cell aplasia. Since CD19 is expressed on all B-cell populations, both cancerous and non-cancerous, CD19 CAR-T cells eliminate both populations. Despite this, patients who receive CD19 CAR-T cells can continue to live with relatively minimal life disruptions with appropriate supportive care [54].

## MECHANISMS OF RESISTANCE TO CAR-T CELL THERAPY IN NHL

Resistance to CAR-T therapy can be categorized as either CAR-T cell-extrinsic or -intrinsic mechanisms; the former encompasses host tumor environment while the latter relates to CAR-T functional changes. Here, we provide an overview of extrinsic and intrinsic mechanisms of CAR-T resistance, including strategies being used to overcome CAR-T resistance.

## EXTRINSIC MECHANISMS OF RESISTANCE TO CAR-T CELL THERAPY

After infusion, CAR-T cells face numerous extrinsic mechanisms of resistance. Most notable of these challenges is the loss of the ability to recognize the tumor antigen and immunosuppression mediated by the tumor microenvironment. Modulation or loss of target antigen expression ultimately eliminates the ability of CAR-T cells to directly kill tumor cells. In approximately 10% to 20% of patients treated with CD19 CAR-T cells within 3 to 6 months of treatment, a CD19^-^ tumor population emerges [54–58]. Due to this phenomenon, patients are screened for target antigen expression prior to receipt of CAR-T cell therapy. Mechanisms of CD19 modulation include deleterious mutations, alternative splicing, lineage switching (in leukemia), and disruption of CD19 transport to the cell surface [59–61]. In all cases, the result is the survival of tumor cells unable to be recognized by the CAR-T cells. Proposed strategies to overcome the loss of CD19 expression largely consist of utilizing additional target antigens such as CD20, CD22, or CD123 in the design of the treatment. Preclinical experiments utilizing co-transduced CD19 and CD22 CAR-T cells or combination CD19 and CD22 CAR-T cells have been promising, with *in vivo* testing showing the ability to eliminate PDX xenografts with or without prior CD19 CAR-T cell therapy [62]. Accordingly, this strategy is now being evaluated in several ongoing clinical trials (eg, NCT03330691; NCT03241940; NCT03233854; NCT03448393; NCT03289455). Another proposed strategy to overcome the loss of target antigen expression is to utilize CAR-T cells that target multiple tumor-specific antigens. One example of this is the work of Schneider et al who described trispecific duoCAR-T cells that concurrently target CD19, CD20, and CD22. *In vivo* and *in vitro* studies of the trispecific duoCAR-T demonstrated the ability to target antigen-heterogeneous mixtures of tumor cells [63]. Clinical trials are currently in progress to evaluate the potential of this approach, and it will be of great interest to appreciate if targeting multiple antigens simultaneously can help improve patient outcomes. Epitope spreading is another method suspected to overcome the loss of target antigen expression where the endogenous immune system is recruited by CAR-T cells to eliminate antigen-negative clones and is well-described in immune checkpoint blockade therapies.

In addition to target antigen modulation, another branch of mechanisms that promote CAR-T cell resistance is immunosuppression via the tumor microenvironment. Tumor cells often express or secrete soluble mediators such as IL-10, TGF-β, and prostaglandin E2 that dampen the anti-tumor response or polarize immune populations toward anti-inflammatory phenotypes, yielding T regulatory cells, M2 macrophages, and myeloid-derived suppressor cells [64, 65]. These cell types, in turn, produce IL-10 and TGF-β to further inhibit the anti-tumor response while supporting angiogenesis and tumorigenesis [66–68]. Additionally, MDSCs deplete L-arginine, a necessary metabolite for T-cell proliferation, from the tumor microenvironment via the expression and secretion of arginase-1 and nitric oxide synthase 2. Solid tumors provide the greatest challenge to CAR-T cell therapy as it also requires CAR-T cell infiltration of the tumor and survival in hypoxic and metabolically hostile conditions. To address these mechanisms, more work is necessary to identify the frequency of relevant factors present in individual NHL subtypes. Strategies to “armor” CAR-T cells against the hostile tumor microenvironment include the incorporation of pro-inflammatory cytokines (IL-12), co-stimulatory ligands (4-1BBL; CD40L), or dominant negative receptors (TGF-β) into the CAR construct [69, 70].

## INTRINSIC MECHANISMS OF RESISTANCE TO CAR-T CELL THERAPY

In addition to the hostility of the tumor microenvironment, CAR-T cell therapies are often limited by failure of cells to replicate and carry out effector functions *in vivo*. Two major such mechanisms are T-cell senescence and exhaustion.

Senescent T cells are terminally differentiated cells that retain their cytotoxic and effector functions but lose the ability to divide and clonally expand on stimulation with antigen. While senescence can occur through telomere-dependent or -independent mechanisms, activated T cells produce telomerase and thereby resist telomere-dependent senescence. Moreover, many T cells in the cancer setting are shown to not have critically short telomere length [71]. Telomere-independent senescence takes place through the p38 pathway, which blocks autophagy and recycling of damaged mitochondria. This leads to increased reactive oxygen species (ROS) production and subsequent DNA damage, which, in turn, acts as positive feedback for the p38 pathway. Induction of the p38 pathway eventually leads to cell cycle arrest through p16 and p53 expression.

Senescent T cells are typically identified by a CD28^-^, CD27^-^, CD57^+^, and KLRG1^+^ phenotype [71, 72]. CD28, the costimulatory molecule needed for naive T-cell activation, is associated with downstream signaling pathways partly responsible for IL-2 production [73]. CD27 promotes clonal expansion through a signaling complex with CD70 [74]. As such, senescent T cells lack the ability to proliferate and produce IL-2. CD57 and KLRG1 expression correspond with terminally differentiated CD4^+^ and CD8^+^ T cells, which retain the ability to secrete IFN-γ and TNF-α and express cytotoxic proteins [75].

In contrast with senescence, T-cell exhaustion is characterized by diminished effector functions, resulting from both chronic antigen stimulation and ineffective priming. Exhausted T cells are identified by the expression of inhibitory receptors, including PD-1, CTLA-4, TIM-3, and LAG-3, and decreased functional markers, including cytotoxicity, cytokine production, and proliferation. Currently, a strategy for ameliorating T-cell exhaustion is treatment with immune checkpoint inhibitors. This approach does not reverse the actual state of exhaustion but instead drives proliferation of non-exhausted T-cell populations by preventing ligand-receptor interactions with inhibitory receptors.

A recent study by our group showed that a second-generation CD19 CAR-T cell displayed an RNA-seq profile of dysfunction and exhaustion with an increased expression of TIGIT and PD-1 on the cell surface. This study further demonstrated that the combination of the CD19 CAR with a monoclonal TIGIT-blocking antibody can lead to enhanced CAR-T cell function and improved efficacy against NHL [15]. Interestingly, results from another study that combined CD19 CAR-T cells with a PD-1 specific VHH domain of an anti-PD-1 nanobody to block PD-1 did not show improvement in NHL outcome and, on the contrary, it significantly reduced survival and diminished cytotoxicity [76]. Therefore, the use of an anti-TIGIT but not of anti-PD-1 antibody may have a unique positive effect in NHL mouse models and the potential to be successfully applied to NHL patients.

A recent study correlated the presence of exhaustion markers on CD8^+^ CAR-T cells with poorer responses to treatment in B-cell malignancies [77]. In non-responding patients, a larger proportion of CD8^+^ CAR-T cells were positive for CD57 and CD39 than in treatment-responsive patients. These CD57^+^CD39^+^ CAR-T cells exhibited increased PD-1 and LAG-3 expression, confirming their phenotype of exhaustion. Thus, the association between exhausted CD8^+^ CAR-T cells and poor therapeutic response warrants further investigation. Specifically, it may be worthwhile to screen for and utilize CD8^+^ CAR-T cells without exhaustion markers in treatment.

## CONCLUSION

In this review, we highlight the use of CAR-T cell therapy in NHL patients. CAR-T cell therapy is an immunotherapy that drastically improves T cell cytotoxic activity by genetically engineering T cells with receptors against an antigen specific to cancer cells without requiring MHC peptide presentation [23–26]. CAR-T cells typically consist of an antigen binding domain, hinge sequence motif, a transmembrane domain, and one or more intracellular signaling domains [30, 31]. While there are currently 4 generations of CAR products, most clinical trials are currently utilizing second-generation CAR constructs, such as ZUMA-7 and TRANSFORM, and have displayed promising results. Notably, patients have experienced serious adverse effects from CAR products, such as cytokine release storm, due to rapid activation and expansion of T cells and neurotoxicity. However, recent clinical strategies have mitigated the morbidities and mortality associated with these side effects. In addition to adverse side effects, resistance to CAR-T cell therapies has also been shown through both extrinsic and intrinsic mechanisms. Extrinsic resistance has been demonstrated by modulation or loss of target antigen expression and immunosuppression via the tumor microenvironment. Intrinsic mechanisms include T-cell exhaustion and senescence. Despite these pitfalls, we remain optimistic for enhancing the effectiveness of CAR-T cell therapies in the future.
